# Hierarchy Graph Based Barrier Coverage Strategy with a Minimum Number of Sensors for Underwater Sensor Networks

**DOI:** 10.3390/s19112546

**Published:** 2019-06-04

**Authors:** Juan Chang, Xiaohong Shen, Weigang Bai, Ruiqin Zhao, Bin Zhang

**Affiliations:** 1 School of Marine Science and Technology, Northwestern Polytechnical University, Xi’an 710072, China; wgbai@mail.nwpu.edu.cn (W.B.); rqzhao@nwpu.edu.cn (R.Z.); 2 Department of Basic Sciences, Air Force Engineering University, Xi’an 710072, China; KGDzhangbin@163.com

**Keywords:** underwater sensor network, barrier coverage, coverage graph

## Abstract

Underwater sensor networks (UWSNs) based barrier coverage is increasingly important for intrusion detection due to the scarcity of underwater sensor resource. To improve UWSNs’ detection performance and prolong their lifetime, an efficient barrier coverage strategy is very important. In this paper, a novel concept: hierarchy graph is proposed. Hierarchy graph can make the network’s topology more clarity. In accordance with the hierarchy graph, 1-barrier coverage algorithm and *k*-barrier coverage algorithm are presented to construct the barrier with less sensors for higher energy efficiency. Both analytical and simulation studies demonstrate that the proposed algorithms can provide high detection probability and long lifetime for UWSNs.

## 1. Introduction

**Figure 1:**
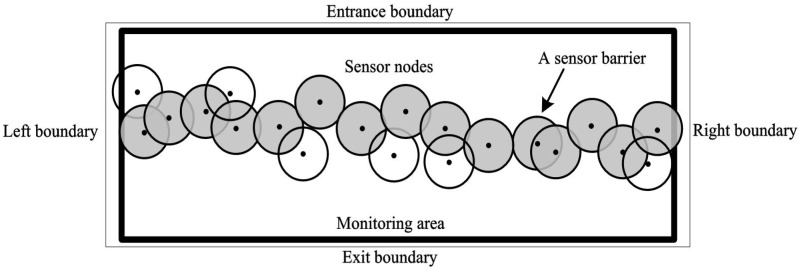
An illustration of the barrier coverage in a monitoring area.

Advances of underwater sensor networks (UWSNs) in providing unprecedented capabilities for surveillance monitoring have motivated the deployment of UWSNs for a broad range of applications such as oceanographic data collection, environmental and pollution monitoring [1], disaster prevention, assisted navigation, intrusion detection [2] and border monitoring [3]. There is a significant interest in intrusion detection for environmental and safety reasons [4]. Network coverage plays an important role in those research fields [5,6]. The full coverage is usually used to cover every point of the monitoring area by at least one sensor. To reduce the deployment cost and the computational complexity, much attention has been paid to the barrier coverage strategies of UWSNs. Such applications require an accurate detection of the moving targets when they are crossing the barrier other than keeping track on the moving targets at every point in the monitoring area. Hence, a full coverage of monitoring area is not needed, and the barrier with a relative small number of sensors may be able to discover the targets. Figure 1 gives an illustration of the barrier coverage in a monitoring area. The entrance boundary and exit boundary are the borders that intruders turn up and leave off from the monitoring region. For efficient placement of sensors, sensors are usually scattered in the monitoring area by Aircraft, submarines or ships, namely, random deployment. In this paper, we will study how to select sensors from the scattered sensors to construct barriers in random deployment.

In harsh underwater environments, the battery power of sensors is limited and usually batteries cannot be recharged because solar energy cannot be exploited. Hence, improving the energy efficiency has become an important challenge of UWSNs. In order to reduce the power consumption, quite a few works give attention to adopt mobile sensors to fill in barrier gaps with minimum moving cost [7,8,9,10,11,12]. In [7], the critical condition is derived to estimate whether barrier gaps may exist. An energy-efficient barrier repair algorithm is proposed to minimize the maximum sensor moving distance with mobile sensors. In [8], a cluster-based directional barrier graph is proposed to model the barrier coverage formation problem and a greedy movement algorithm for heterogeneous wireless sensor networks (WSNs) is derived to efficiently schedule different types of mobile sensors to different gaps while minimizing the total moving cost. In [9], the problem how mobile sensors can be efficiently relocated to achieve k-barrier coverage is studied based on linear programming, which is formulated as 0–1 integer linear programming (ILP). Then, the integrality and complicated constraints of the formulation is relaxed and a special model known as RELAX-RSMN is constructed to solve the relaxed 0–1 ILP through linear programming with a totally unimodular constraint coefficient matrix. In [11], a dynamic intruder detection scheme is proposed. The mobile sensors move smartly to dynamically change the topology and minimum exposure path of whole network. The smart intruders can be detected with a high probability. However, in stationary UWSNs, we try to successfully construct barriers at the first time, without filling barriers’ gaps.

It is well known that the energy constraint of sensors determines the lifetime of the network directly. In UWSNs, the sensors powered by batteries have very limited energy. Recent research has been devoted to optimizing a sleep-wakeup scheme for networks to improve the energy efficiency [13,14,15,16]. In [13], the concept of local barrier coverage is introduced to develop a novel sleep-wakeup algorithm for maximizing the network lifetime. In [14], an optimal sleep-wakeup algorithm is proposed for non-disk sensing regions and heterogeneous sensing regions. This algorithm provides an optimal solution for heterogeneous lifetime case. In [15], an optimal algorithm is also proposed not only for equal lifetime, but also for the case when sensor lifetimes are different. This algorithm can be used to maintain not just barrier coverage, but fault tolerant connectivity, while maximizing the network lifetime. In addition, in [17], an effective algorithm is proposed to construct barriers with low message complexity to preserve the energy in each node and with minimum cost to extend the lifetime of the wireless sensor network.

The above works focus more on saving UWSNs’ energy efficiency. Nevertheless, the detection performance has not been fully explored. In order to take account of energy efficiency and detection performance simultaneously, we need to focus on constructing barriers with a minimum number of sensors. The fewer sensors that are used to construct a barrier, the more barriers that could be constructed and the higher detection probability that could be achieved. Moreover, more barriers can guarantee longer network lifetime as barriers can work alternatively. In the case of limited underwater sensor resource, it is efficient to use as few sensors as possible. However, so far, little effort has been made on constructing barriers with a minimum number of stationary sensors after initial random deployment.

In this paper, we study the barrier coverage strategy in random deployment with a minimum number of stationary sensors. The major intellectual contributions of this paper are as follows:

- A novel conception of hierarchy graph is defined. Consequently, we present a strategy to obtain barriers with a minimum number of required sensors based on hierarchy graph (MNHG). After constructing the coverage graph according to the distances between the adjacent sensors, the coverage graph can be graded to some hierarchies. Based on the hierarchy graph, we can find the nodes with a minimum number to construct barriers.
- During our research, we derive the algorithm of 1-barrier coverage with a minimum number of sensors (*1-MNHG*) and the algorithm of *k*-barrier coverage with a minimum number of sensors (*k-MNHG*).

This paper is organized as follows. In Section 2, some related works are briefly reviewed. We describe the sensors’ coverage model and UWSNs’ model in Section 3. In Section 4, 1-MNHG and *k*-MNHG algorithm are proposed. In Section 5, the simulation results are shown. Finally, the main conclusions are drawn in Section 6.

## 2. Related Work

When the number of randomly deployed sensors is unchanged, constructing the barrier using less sensors, we can increase the probability of constructing more barriers. Accordingly, there are more barriers working alternately to prolong the UWSNs’ lifetime. If more barriers work at the same time, the target’s detection probability in UWSNs can also be improved.

Some works give attention to adopt the minimum number of mobile sensors to construct barriers [18,19,20]. In [18], Wang Zhibo et al. introduce a novel concept of weighted barrier graph and propose an optimal solution for the minimum number of mobile sensors to form k-barrier coverage when only the stationary sensors have been deployed. In [19], a novel full-view covered model of mobile camera sensors is proposed to construct barriers by a grid-based strategy with a minimum number of camera sensors.

In order to reduce the number of stationary sensors required to construct a barrier and hence to prolong the network lifetime, in [21], splitting sensor deployment into multiple rounds is proposed to reduce the number of sensors required to provide guaranteed barrier coverage. Two practical solutions are proposed: (i) the two round minimax solution and (ii) the pilot deployment solution, to deal with realistic situations when the knowledge about the deviation of sensors’ residence points is not fully available. However, those solutions only apply to the 0/1 sensing model, not more realistic coverage models.

In addition, Mostafaei et al. adopt learning automata to construct barriers using as few sensors as possible [22,23,24]. In [23], Mostafaei et al. model the barrier coverage problem based on stochastic coverage graph and propose a distributed learning automata-based method to find the minimum possible number of nodes in each barrier to the stochastic barrier coverage problem. Nevertheless, their learning automata-based algorithms only find the near optimal solution, that is to say, we cannot construct the barrier with a minimum number of stationary sensors using their algorithm.

Existing barrier coverage strategies can be divided into two categories: (i) weak barrier coverage, and (ii) strong barrier coverage [25]. As shown in Figure 1, strong barrier coverage guarantees that the moving target along the arbitrary path can be detected, and the weak barrier coverage requires the detection of the moving target along the perpendicular path. The minimum number of stationary sensors required by the strong barrier coverage has been invariably difficult. In [26], a distributed algorithm that constructs sensor barriers in long-strip areas of irregular shape is studied. The algorithm separates the region into segments and then connects small segments of horizontal barriers and vertical barriers to form several barriers across the original region. The close-to-optimal solutions based on directional non-overlapping model is presented. In [27,28], a polynomial time algorithm based strong barrier coverage is proposed. The virtual node is introduced to capture the geographical relationships among sensors. In addition, a directional barrier graph is obtained quickly. According to the barrier graph, the barrier coverage over a belt region can be constructed adopting the Dijkstra shortest path algorithm [29]. In [18], a weighted barrier graph and an optimal solution based on greedy search to find k vertex-disjoint paths are investigated. The k-barrier coverage algorithm based on the weighted graph requires each sensor to know the information of others. In the above works, various algorithms are proposed on the basis of the shortest path algorithm. The shortest path algorithm is a classical algorithm in the graph theory, which is typically implemented in sensor networks with hundreds of nodes. The complexity of that algorithm is Ω(mn), where *n* is the number of vertices and *m* is the number of edges. The Dijkstra algorithm iterates through all vertices in predefined order. In each iteration, a vertex with a minimum distance is found among those which have not been previously processed, which makes the computational complexity is high. Furthermore, the number of the required sensors constructing the barrier coverage is large.

In [30], the UT(upper triangle)-adjacency matrix-based algorithm is proposed to construct the barrier coverage. The upper triangle of the adjacent matrix is used to construct the barrier coverage based on the dynamic programming. The adjacency matrix based on the graph theory expresses the relationship of vertices in the graph. In the barrier coverage, the adjacency matrix has its own unique characteristics. Those characteristics impose certain regularities on searching the adjacency matrix’s upper triangle. The number of sensors constructing the barrier coverage is small. However, it is also hard to construct barriers with a minimum number of sensors.

## 3. Models and Problem Statement

### 3.1. Sensor Coverage Model

In this paper, an energy attenuation disk coverage model is utilized. It is more applicable for the UWSNs. When the acoustic signal propagates in underwater environments, the energy of the received acoustic signal is affected not only by the transmission distance, but by the underwater noise and the absorption loss of acoustic wave. When we consider the underwater noise and the absorption loss of acoustic wave, the acoustic signal propagating in an underwater environment follows the passive sonar equation [31]:

(1) SL−TL=NL−DI+DT,

where *SL* represents emission source level, *TL* represents transmission loss and *NL* represents noise level. Their units are all decibel (dB). Assuming spherical expansion of underwater acoustic signals, *TL* is expressed as

(2) $$TL=10\mathrm{lg}\frac{d^{2}\left(s,z\right)}{r_{ref}}+\alpha d\left(s,z\right),$$

where d(s,z) shows Euclidean distance between the sensor *s* and the target *z*, and $r_{ref}$ is the reference distance, which usually takes a value of 1. αd(s,z) is the absorption loss of the acoustic signal. α is absorption coefficient, its unit is dB/km, which can be expressed as

(3) $$\alpha =\frac{0.102f^{2}}{1+f^{2}}+\frac{40.7f^{2}}{4100+f^{2}}+3.06\times 10^{-4},$$

where *f* is working frequency. *NL* is expressed as

(4) $$NL=SNL+10\mathrm{lg}B_{f},$$

where *SNL* is noise spectrum level over 1 Hz bandwidth, and $B_{f}$ is receiving bandwidth. In addition, *DI* and *DT* represent receiving directivity index and the receiver’s detection threshold, respectively. In this coverage model, we are focused primarily on the received signal’s energy, the receiver’s detection threshold *DT* is not considered. In this paper, all the sensors are assumed to be homogeneous and isotropic in UWSNs, which determines DI=0.

The received signal’s energy is

(5) $$E\left(s\right)=10^{\frac{SL-TL-NL}{10}},$$

where E(s) is received signal’s energy in the spatial point *z*. The energy attenuation disk coverage model in the UWSN is described as Equation (5). From Equations (1) and (2), we can see that E(s) is inversely proportional to the square of the distance d(s,z). Hence, the sensor’s received Signal-to-noise ratio (SNR) will change with the distance between the sensor and the target. It is worth noting that the sensing radius of the sensor is not fixed in this energy attenuation disk coverage model. We can construct barriers using the radius with the appropriate value, which we call constructing radius.

### 3.2. Detection Models

There are two common detection models: individual detection model and cooperative detection model [32]. In this paper, we adopt an individual detection model to detect the target based on energy detection. The false alarm probability $P_{f}$ is defined as the probability that the sensor falsely decides that the target is present when the target is absent. The detection probability $P_{d}$ is defined as the probability that the sensor correctly decides that the target is present when the target is present.

Hypothesize that $H_{1}$ represents that the target is present and $H_{0}$ represents the target is not. Set $E_{p}=10^{\frac{SL}{10}}$, $w_{i}=10^{\frac{NL}{10}}$. Then, taking into account the energy attenuation disk coverage model, the readings $x_{i}$ at the sensor $s_{i}$ are given by

(6) $$\left\{\begin{array}{c} H_{0}:x_{i}=w_{i},\\ H_{1}:x_{i}=\frac{E_{p}}{d_{i}^{2}}+w_{i}, \end{array}\right.$$

where $d_{i}=d\left(s_{i},z\right)$ and $w_{i}$ is the measurement noise. We suppose that the noise $w_{i}$ follows a Gaussian distribution with zero mean and variance $\sigma^{2}$. Under $H_{0}$ condition, $x∼N(0,\sigma^{2})$, and under $H_{1}$ condition, $x∼N(0,\sigma_{s}^{2})$. The number of sampling points for each sensor node is M. The test statistics for energy detection is

(7) $$T\left(x\right)=\left\{\begin{array}{c} \frac{1}{\sigma^{2}}\sum_{m=1}^{M}w_{i}^{m},H_{0},\\ \frac{1}{\sigma_{s}^{2}+\sigma^{2}}\sum_{m=1}^{M}\left(\frac{E_{p}}{{\left(d_{i}^{m}\right)}^{2}}+w_{i}^{m}\right),H_{1}. \end{array}\right.$$

We also assume that the detection threshold is γ. When

(8) $$T\left(x\right)\overset{H_{1}}{\underset{H_{0}}{⋛}},\;\gamma$$

a sensor makes its detection decision. When the number of sampling points is large enough and a target is active at the space point *z*, the detection probability $P_{d}$ of the sensor $s_{i}$ is given by

(9) $$\begin{array}{ll} P_{d}^{i} & =Pr[T\left(x\right)\geq \gamma ;H_{1}]\\ & =Q_{\chi_{M}^{2}}\left(\frac{\gamma }{\frac{E_{p}}{{\left(d_{i}^{2}\right)}^{2}}\sigma_{s}^{2}+\sigma^{2}}\right), \end{array}$$

where Q(•) is the *Q*-function defined by

(10) $$Q_{\chi_{M}^{2}}=\int_{x}^{+\infty }p(t)dt.$$

### 3.3. UWSNs’ Model

The underwater sensor network(UWSN) consists of *N* sensors, the set of sensors is $\left\{s_{1},s_{2},\cdots ,s_{N}\right\}$, where $s_{i}$ expresses the *i*th sensor. Sensors are deployed randomly in a two-dimensional belt area $A_{2-dim}=\left[0,L\right]\times \left[0,W\right]$, where *L* and *W* represent the length and the width of the belt region, respectively.

To facilitate the discussion in this paper, several assumptions are made as follows:

- The isotropic sensors in the UWSN are homogeneous and the sensing radius of the sensors is $R_{s}$.
- All sensor nodes can calculate their relative positions to each other through many localization methods [33,34,35].
- The UWSN has connectivity owing to underwater wireless communication technology [36].
- All the sensors are stationary and their positions remain unchanged.
- During crossing the monitor region, the radiated energy of the target remains unchanged.

### 3.4. Problem Statement

Suppose *S* is the set of sensors that constructs a barrier, and the sensors $s_{i}\left(x_{i},y_{i}\right)\in S$, $s_{j}\left(x_{j},y_{j}\right)\in S$, where $\left(x_{i},y_{i}\right)$, $\left(x_{j},y_{j}\right)$ is respectively the coordinate of the sensors $s_{i}$ and $s_{j}$. We want to construct a barrier with a minimum number of sensors, which can be described as an optimization problem. In accordance with the definition [1] of the strong barrier coverage, the optimization problem is expressed:

(11) $$\begin{array}{c} \min{\left\|S\right\|}_{0},\\ s.t.\left\{\begin{array}{c} s_{i}\in S,s_{j}\in S,\\ d\left(s_{i},s_{j}\right)\leq 2R_{s},\\ 0\leq x_{i}\leq L,0\leq x_{j}\leq L,\\ 0\leq y_{i}\leq W,0\leq y_{j}\leq W, \end{array}\right. \end{array}$$

where $d(s_{i},s_{j})$ is the Euclidean distance between two sensors $s_{i}$, $s_{j}$. and ${\left|•\right|}_{0}$ expresses the number of elements in a set.

Facing the above NP(non-deterministic polynomial) hard problem, it is very arduous for us get the optimal solution. Researchers often try to get the near optimal solution [22,23,27]. In this paper, we construct barriers by grading the coverage graph to get a more approximate Optimal Solution.

## 4. Basic Idea of Hierarchy Graph Based Barrier Coverage Strategy

**Figure 2:**
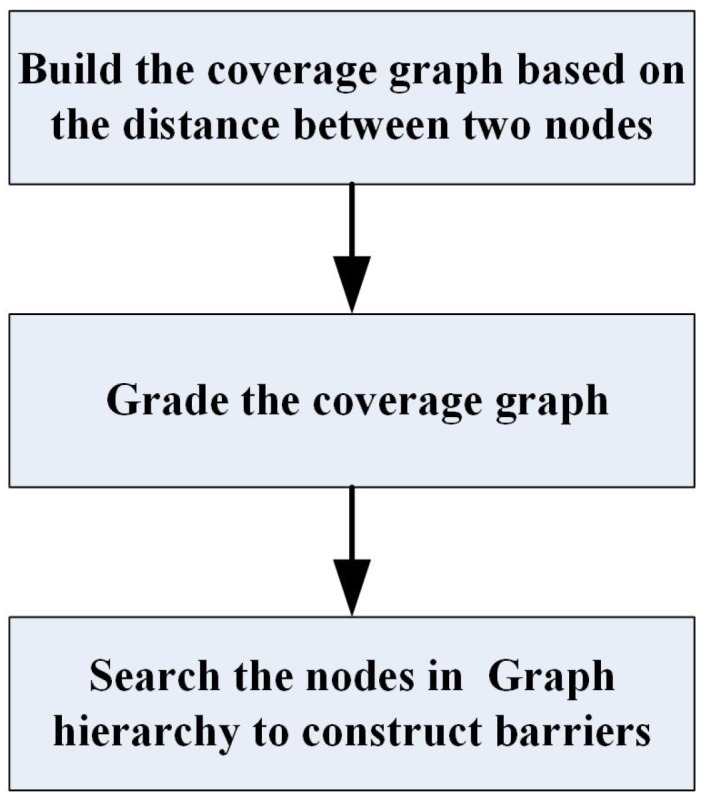
The process of hierarchy graph based barrier coverage strategy.

In this section, the conception of hierarchy graph is introduced to solve the problem of constructing the barrier coverage with a minimum number of sensors. The process of the strategy is shown as Figure 2. Adopting the strategy, we can also construct strong (or weak) barriers in UWSNs composed of sensor nodes that have different characteristics. It is important to note that this strategy is implemented by a central node, which may be a powerful sensor or an underwater vehicle in UWSNs. Thus, the new strategy is centralized.

### 4.1. Coverage Graph

**Figure 3:**
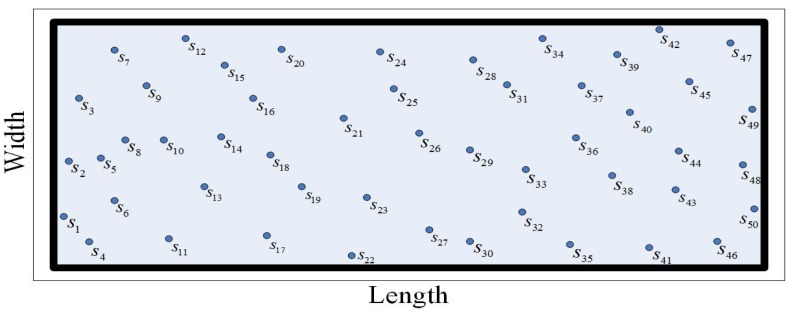
The UWSN in random deployment.

Consider the lower line of the monitoring region as the horizontal axis and the left line of the monitoring region as the vertical axis. For ease of the following discussion, the abscissa of all the randomly deployed sensors is in the ascending sort order. The leftmost sensor is $s_{1}$ and the rightmost sensor is $s_{N}$. A UWSN randomly deployed is shown in Figure 3.

Obtaining the distance between sensors is the basic operation for WSNs’ coverage and transmission [37,38]. In this strategy, the coverage graph is built based on the distance between sensors.

**Definition** **1.**  *If the horizontal projection of two sensors’ sensing area overlap, these two sensors are weakly connected.*

In the random deployment, the weak connection needs that the projections of the two sensors’ sensing region on the horizon intersect. The two sensors satisfy:

(12) $$d_{x}\left(s_{i},s_{j}\right)=\left|x_{j}-x_{i}\right|\leq 2R_{S},$$

where $\left(x_{i},y_{i}\right)$ and $\left(x_{j},y_{j}\right)$ are two sensors’ position coordinates, respectively. $d_{x}\left(s_{i},s_{j}\right)$ is the horizontal distance between two sensors.

**Definition** **2.**  *If two sensors’ sensing area overlap, these two sensors are strongly connected.*

The strong connection needs intersection of the two sensors’ sensing region, that is to say, the Euclidean distance of the two nodes must satisfy:

(13) $$d\left(s_{i},s_{j}\right)=\sqrt{{\left(x_{j}-x_{i}\right)}^{2}+{\left(y_{j}-y_{i}\right)}^{2}}\leq 2R_{s}.$$

The coverage graph consists of connected sensors. In accordance with the above conditions, we can build the coverage graph for the strong barrier and the weak barrier, respectively.

**Figure 4:**
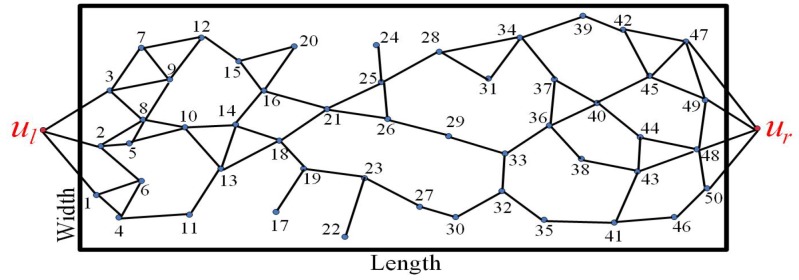
The coverage graph of the UWSN shown in Figure 3 for the strong barrier.

**Definition** **3.**  *Let* G(V,E) *denote the coverage graph, V and E represent its vertex set and edge set, respectively [18]. In addition, we add two virtual nodes* $u_{l}$ *and* $u_{r}$ *. It is worth noting that the virtual node* $u_{l}$ *is only connected with the node whose distance with the left boundary is less than* $R_{s}$ *. Similarly, the virtual node* $u_{r}$ *is only connected with the node whose distance with the right boundary is less than* $R_{s}$ *. The coverage graph of the UWSN shown in Figure 3 for the strong barrier is presented in Figure 4.*

### 4.2. Grade the Coverage Graph

Constructing barrier coverage, especially strong barrier coverage with a minimum number of sensors, is very difficult due to the intricate topological relationships among sensors. In [39], an M-stage network to optimize the performance of their scheme is proposed. Stage network is derived by grading the network according to certain conditions, which makes network’s hierarchies clearer. Motivated by [39], we proposed a new conception: hierarchy graph in this paper. Hierarchy graph is a graded coverage graph according to certain conditions, which is described in Definition 4:

**Definition** **4.**  *If the structure of the coverage graph* G(V,E) *satisfies the following conditions:*

1. *If*i≠j(i,j=1,2,⋯,T)*,*${\mathrm{HI}}_{i}⋂{\mathrm{HI}}_{j}$ = ⌀ *and HI =* ${⋃}_{t=1}^{T}{\mathrm{HI}}_{t}$*, where* ${\mathrm{HI}}_{i}$ *expresses a hierarchy in the coverage graph.*
2. *Only two adjacent hierarchies’ nodes (except for the case that one hierarchy is connected with* $u_{l}$ *or* $u_{r}$ *) or two nodes in the same hierarchy can reach each other.*
3. $\forall s(s\in {\mathrm{HI}}_{t})$ *is invariably connected with some nodes in* ${\mathrm{HI}}_{t-1}$ *(or* $u_{l}$ *) and* ${\mathrm{HI}}_{t+1}$ *(or* $u_{r}$ *) at the same time.*

G(V,E) is called hierarchy graph. ${\mathrm{HI}}_{1},{\mathrm{HI}}_{2},\cdots ,{\mathrm{HI}}_{T}$ are called barrier hierarchies. ${\mathrm{HI}}_{t}$(1≤t≤T) is the *t*th barrier hierarchy in hierarchy graph. Based on the conditions in Definition 4, hierarchy graph can clarify the topological relationships among nodes to facilitate the realization of barrier coverage with a minimum number of sensors.

According to Definition 4, each node must be directly connected with the nodes in its lower hierarchy and its higher hierarchy at the same time. We grade the graph from $s_{1}$ to $s_{N}$. The closest hierarchy to $u_{l}$ is the lowest hierarchy (${\mathrm{HI}}_{1}$), and the closest hierarchy to $u_{r}$ is the highest one (${\mathrm{HI}}_{T}$). The coverage graph clarifies the connection relationship among sensors. Based on the clear connection relationship, the nodes connected with $u_{l}$ consists of ${\mathrm{HI}}_{1}$. When finding nodes connected with ${\mathrm{HI}}_{t}$ to form ${\mathrm{HI}}_{t+1}$, we need to remove some nodes from ${\mathrm{HI}}_{t}$, which is only connected with one adjacent hierarchy ${\mathrm{HI}}_{t-1}$.

**Figure 5:**
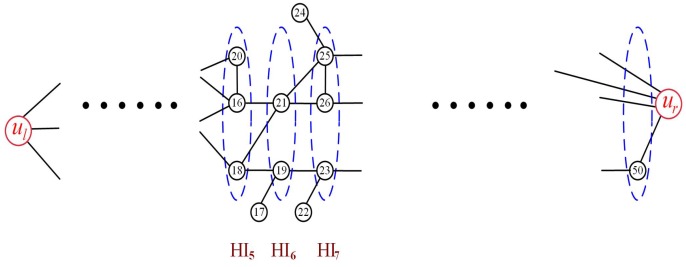
Partial unfinished hierarchy graph.

**Figure 6:**
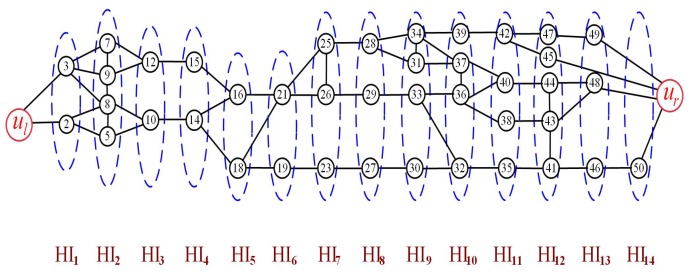
The hierarchy graph.

A partial unfinished hierarchy graph is shown as Figure 5. Some nodes only connected with their one adjacent hierarchy must be removed. Thus, not all the nodes in the network are in the hierarchy graph. For example, the nodes marking 17, 20, 22, 24 must be removed. Those removed nodes are futile to construct the barrier. However, they can be used to repair the coverage hole caused by the node mobility and the ocean currents. A finished hierarchy graph is shown as Figure 6. The coverage graph has graded 14 hierarchies. The nodes marking 45, 48, 49, 50 are all connected with $u_{r}$, but not all of them belong to the highest hierarchy (${\mathrm{HI}}_{14}$).

Based on the hierarchy graph, four deductions are shown as follows:

A. Assume that a coverage graph can be graded as ${\mathrm{HI}}_{1},{\mathrm{HI}}_{2},\cdots ,{\mathrm{HI}}_{T}$. If there is minimal t(1≤t≤T), which satisfies that there exists at least one node in ${\mathrm{HI}}_{t}$ connected with $u_{r}$ directly and with nodes in ${\mathrm{HI}}_{t-1}$ simultaneously, then, *t* is the minimum number of the nodes constructing a barrier. ${\mathrm{HI}}_{1},{\mathrm{HI}}_{2},\cdots ,{\mathrm{HI}}_{T}$ are called barrier hierarchies. We also call $\left\{{\mathrm{HI}}_{1},{\mathrm{HI}}_{2},\cdots ,{\mathrm{HI}}_{t}\right\}$ the minimum set of barrier hierarchies. *t* is the number of barrier hierarchies.

**Proof.**  Assume that *t* is not the minimum number of the nodes constructing a barrier, there are two cases, $\exists t^{\prime }>t$ is the minimum number and $\exists t^{\prime }<t$ is the minimum number:

- If $t^{\prime }\left(t^{\prime }>t\right)$ is the minimum number, there is no node connected with $u_{r}$ directly in ${\mathrm{HI}}_{t}$. This is contradictory to the assumption in Conclusion A.
- If $t^{\prime }\left(t^{\prime }<t\right)$ is the minimum number, there is at least one node in ${\mathrm{HI}}_{t}$ connected with $u_{r}$ directly. This is contradictory to the assumption in Conclusion A.

 □

B. At least one barrier can be built in the hierarchy graph. In other words, as long as the hierarchy graph is constructed successfully, the probability of failure to construct a barrier is 0.

**Proof.**  Assuming that one barrier cannot be built, there must be one node connected with only one adjacent hierarchy, which is contradictory to the condition 3 in Definition 4. □

C. If the UWSN is graded as ${\mathrm{HI}}_{1},{\mathrm{HI}}_{2},\cdots ,{\mathrm{HI}}_{T}$ and $\left\{{\mathrm{HI}}_{1},{\mathrm{HI}}_{2},\cdots ,{\mathrm{HI}}_{t}\right\}$ is the minimum set of barrier hierarchies which can construct *k*-barrier coverage. Then, $k\leq \min\{|{\mathrm{HI}}_{1}{|}_{0},|{\mathrm{HI}}_{2}{|}_{0},\cdots ,|{\mathrm{HI}}_{t}{|}_{0}\}$. (${\left|•\right|}_{0}$ expresses the number of elements in a set.)

**Proof.**  Assume $k>\min\{|{\mathrm{HI}}_{1}{|}_{0},|{\mathrm{HI}}_{2}{|}_{0},\cdots ,|{\mathrm{HI}}_{t}{|}_{0}\}$ and the coverage graph is graded *T* hierarchies (t≤T). Thus, there is only one situation in which there is at least one hierarchy ${\mathrm{HI}}_{m}$ not used for *k*-barrier coverage. This means that some nodes in the higher hierarchy ${\mathrm{HI}}_{m+1}$ are connected with those in the lower hierarchy ${\mathrm{HI}}_{m-1}$ directly, which is contradictory to the condition (3) of hierarchy graph. □

D. If there are *p* nodes connected with $u_{l}$ and *q* nodes connected with $u_{r}$. Then, *k*-barrier coverage is constructed, k≤min{p,q}.

**Proof.**  Assume that the constructed $k^{\prime }$-barriers satisfy $k^{\prime }>\min\left\{p,q\right\}$. There are at least $k^{\prime }$ nodes connected with $u_{l}$ or $u_{r}$, respectively, which is contradictory to the assumption in Deduction A. □

### 4.3. Searching Nodes to Construct Strong Barriers

The 1-MNHG algorithm shown in Algorithm 1 can be used to construct 1-strong barrier coverage. The input and output variables are introduced in the first two lines, where $s_{i}\left(x_{i},y_{i}\right)$, $R_{s}$, ${\mathrm{HI}}_{t}$, ${\mathrm{HI}}_{t_{0}}$, Bar mean the *i*th sensor’s position coordinates, constructing radius, a barrier hierarchy, the highest hierarchy in the minimum set of barrier hierarchies and the set of sensors constructing a barrier respectively. The process of searching nodes to construct a barrier consists of three steps shown in Algorithm 1.

Step 1: Construct the coverage graph and grade it in accordance with the description in Section 4.2. ${\mathrm{HI}}_{1}$ is formed from lines 1–5. Select nodes strongly connected with a node in ${\mathrm{HI}}_{t-1}$ in the loop from lines 9–18. Meanwhile, remove the nodes from ${\mathrm{HI}}_{t-1}$ if they are only connected with higher hierarchy and are not connected with the remaining nodes. ${\mathrm{HI}}_{t}$ is formed in the bigger loop from lines 7–19. The hierarchies except ${\mathrm{HI}}_{1}$ are formed in the loop from lines 6–21.

Step 2: Find the minimum number of sensors constructing a barrier. According to Deduction A and C, the highest hierarchy in the minimum set of barrier hierarchies is the minimum number of sensors. Query nodes connected with $u_{r}$ from the highest hierarchy to the lowest one in the loop from lines 23–28. If there are nodes in the hierarchy *t* connected with $u_{r}$, continue to query in the lower hierarchy. If it is not in the hierarchy *t*, stop querying and t+1 is the minimum number.

Step 3: Search the nodes to construct a barrier. The node connected with the former selected sensor is found in ${\mathrm{HI}}_{t}$ in the loop from lines 36–41. A complete barrier is constructed in the loop from lines 35–42.

According to the Deduction B, there must be a node in lower hierarchy connected with the former selected node. Thus, we needn’t go back to higher hierarchy to search nodes again. Moreover, failure to build a barrier will not happen, if the hierarchy graph is built and graded successfully. Following this scheme, the nodes will be found to construct 1 barrier with a minimum number of nodes eventually.

The *k*-MNHG algorithm depicted in Algorithm 2 can be used to construct *k*-strong barrier coverage. According to the definition of *k*-barrier coverage, the different barriers cannot share the same node. In order to construct as many barriers as possible, we cannot guarantee that all the found nodes in *k*-barrier coverage come from hierarchies in the minimum set of barrier hierarchies. We construct each barrier from the lowest hierarchy and the highest hierarchy. The variable initialization is the same as Algorithm 1. *k* is the number of barriers. After constructing and grading the coverage graph in step 1, several barriers are constructed in step 2. It is worth noting that we need to update the hierarchy graph after each barrier is constructed until all the barriers have been constructed. When updating the hierarchy graph, the nodes selected for the former barrier are removed. Meanwhile, the nodes only connected with one adjacent hierarchy are removed. The new hierarchy graph will be achieved. Obviously, the number of sensors in the updated hierarchy graph is less than that in the original hierarchy graph.

**Algorithm 1** 1-MNHG Algorithm
INPUT: $s_{i}\left(x_{i},y_{i}\right)\left(i=1,\ldots ,N\right),R_{s}$. OUTPUT: ${\mathrm{HI}}_{t}\left(t=1,\ldots ,T\right),\;{\mathrm{HI}}_{t_{0}},\;Bar$. step 1: Construct coverage graph and grade it.
1:**for**i=1:N**do**2: **if** $x_{i}\leq R_{s}$ **then**
3:  ${\mathrm{HI}}_{1}\leftarrow s_{i}$;4: **end if**
5:**end for**6:**while**t<N/2**do**7: **for** $i=1:length({\mathrm{HI}}_{t-1})$ **do**
8:  TM=[];9:  **for** $j=(\sum_{H_{1}}^{H_{t-1}}length\left({\mathrm{HI}}_{t-1}\right)+1):N$ **do**
10:   **if** $d\left(s_{i},s_{j}\right)\leq 2R_{s}$ **then**
11:    ${\mathrm{HI}}_{t}\leftarrow s_{i}$;12:    $\mathrm{TM}\leftarrow s_{j}$;13:   **else if** TM is null **then**14:    Remove $s_{i}$ from ${\mathrm{HI}}_{t-1}$;15:   **else**
16:    break;17:   **end if**
18:  **end for**
19: **end for**
20: t=t+1;21:**end while**step 2: Find the highest hierarchy in the minimum set of barrier hierarchies.22:**for**$t=length({\mathrm{HI}}_{T}):1$**do**23: **for** $i=1:length({\mathrm{HI}}_{t})$ **do**
24:  **if** $L-x_{s_{i}}\leq R_{s}$ **then**
25:   $t_{0}\leftarrow t$;26:   break;27:  **end if**
28: **end for**
29: **if** $t_{0}$ is not null **then**30:  break;31: **end if**
32:**end for**step 3: Find the nodes to construct a barrier:33:$Bar\leftarrow {\mathrm{HI}}_{t_{0}}\left(length\left({\mathrm{HI}}_{t_{0}}\right)\right)$;34:$t=t_{0}$;35:**while**t≥1**do**36: **for** $j=1:length({\mathrm{HI}}_{t})$ **do**
37:  **if** $d\left(Bar\left(length\left(Bar\right)\right),s_{j}\right)\leq 2R_{s}$ **then**
38:   $Bar\leftarrow s_{j}$;39:   break;40:  **end if**
41: **end for**
42:**end while**


**Algorithm 2** k-MNHG Algorithm
INPUT: $s_{i}\left(x_{i},y_{i}\right)\left(i=1,\ldots ,N\right),R_{s}$. OUTPUT: ${\mathrm{HI}}_{t}\left(t=1,\ldots ,T\right),\;{\mathrm{HI}}_{t_{0}},\;k,\;Bar$.
step 1 is same to step 1 in Algorithm 1. step 2: 1:$k=\min\left\{|{\mathrm{HI}}_{1}{|}_{0},|{\mathrm{HI}}_{2}{|}_{0},\ldots ,{\left|{\mathrm{HI}}_{T}\right|}_{0},\right\}$2:**for**l=1:k**do**3: **for** $i=1:length({\mathrm{HI}}_{1})$ **do**
4:  **if** $x_{s_{i}}\leq R_{s}$ **then**
5:   $BB\leftarrow s_{i}$
6:  **end if**
7: **end for**
8: $Bar\left(k,1\right)\leftarrow \min\left\{BB(y)\right\}$;9: **for** t=2:T **do**
10:  **for** $i=1:length({\mathrm{HI}}_{t})$ **do**
11:   **if** $d\left(Bar\left(length\left(Bar\left(k,:\right)\right)\right),s_{j}\right)\leq 2R_{s}$ **then**
12:    $BB\leftarrow s_{j}$
13:   **end if**
14:  **end for**
15:  $Bar\left(k,t\right)\leftarrow \min\left\{BB(y)\right\}$;16: **end for**
17: Update the hierarchy graph and remove the selected nodes.18:**end for**


**Figure 7:**
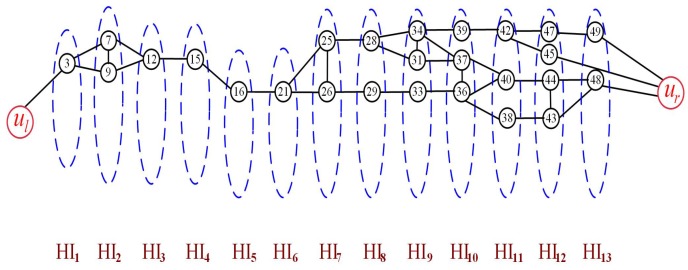
The updated hierarchy graph.

Assume that the number of barriers *k* is equal to 2 shown as Figure 6. There are two nodes marking 2 and 3 in ${\mathrm{HI}}_{1}$ connected with $u_{l}$ directly. Firstly, select the node 2 with the minimum longitudinal coordinate to be the first node in the first barrier, represented as $B_{1,1}$. Secondly, find the nodes in the higher hierarchy ${\mathrm{HI}}_{2}$ connected with $B_{1,1}$ directly and select one with the minimum longitudinal coordinate from them to be the second node in the first barrier, represented as $B_{1,2}$. Following this operation, the remaining nodes in the first barrier can be found to construct the first barrier. Finally, update the hierarchy graph and repeat all of the above operations to construct the other barrier. The updated hierarchy graph is shown as Figure 7. For ease of observation, the position of the nodes in each hierarchy is consistent with their longitudinal coordinates. Based on the update hierarchy graph, we can select nodes to construct the second barrier.

### 4.4. Computational Complexity

Not all the nodes in the network are in the hierarchy graph. Suppose that the number of nodes in hierarchy graph is $N_{h}\left(N_{h}\leq N\right)$ (*N* is the number of sensors in the network) and that the number of hierarchy is $T_{0}$. Then, the average number of nodes in each hierarchy is $\frac{N_{h}}{T_{0}}$.

**Figure 8:**
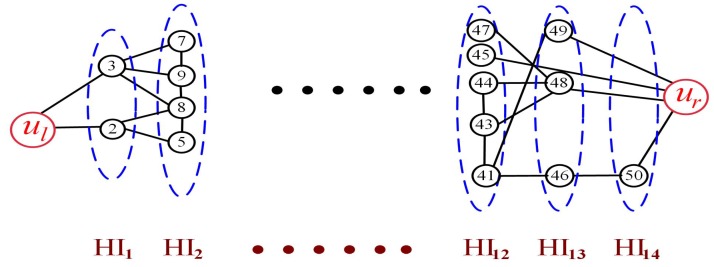
An example of a partial hierarchy graph.

Algorithm 1’s computational complexity consists of two parts. The first part is the computational complexity of grading graph. When forming the hierarchy ${\mathrm{HI}}_{t}$, we need to find nodes connecting with all the nodes in the ${\mathrm{HI}}_{t-1}$. Finding nodes connecting with a node in the ${\mathrm{HI}}_{t-1}$ needs $\frac{N_{h}}{T_{0}}$ computation. Finding nodes connecting with all the nodes in the ${\mathrm{HI}}_{t-1}$ needs $\frac{{N_{h}}^{2}}{{T_{0}}^{2}}$ computation. Forming all the hierarchies needs $\frac{{N_{h}}^{2}}{{T_{0}}^{2}}\times T_{0}$ = $\frac{{N_{h}}^{2}}{T_{0}}$ computation. The second part is the computational complexity of searching nodes to construct a barrier. From Algorithm 1, we can see that we only need to select each node directly connected with the former selected node from its lower hierarchy, after building and grading the coverage graph successfully. If one node has been selected for a barrier, we need to search nodes in its lower hierarchy to select the next node for a barrier. We also suppose the worst case that the next node is selected in the last search from its hierarchy. For example, the node marking 49 has been selected to be the first node in a barrier shown as Figure 8. We have to search all the nodes in its lower hierarchy. Finally, the node marking 41 is selected. In this case, $\frac{N_{h}}{T_{0}}$ computation is needed. If each node for a barrier is selected in the last search from its hierarchy, searching nodes to construct a barrier needs $\frac{N_{h}}{T_{0}}\cdot T_{0}$ = $N_{h}$ computation. As a result, constructing a complete barrier needs $\frac{{N_{h}}^{2}}{T_{0}}+N_{h}$ computation. The computational complexity of 1-MNHG algorithm is $O(N^{2})$.

In Algorithm 2, once a barrier is constructed, the hierarchy graph must be updated. As the number of sensors in the updated hierarchy graph is less than that in the original hierarchy graph. Constructing *k* barriers needs less than $k(\frac{{N_{h}}^{2}}{T_{0}}+N_{h})$ computation. As a result, the computational complexity of *k*-MNHG algorithm is $O(N^{2})$, which is equivalent to the shortest path algorithm and the UT-adjacency matrix-based algorithm.

In this paper, we use the energy attenuation disk coverage model in UWSNs. If we can obtain the coverage graph according to the position relations between directional sensors, we can construct barriers in directional sensor networks using the proposed algorithms. In addition, in our research, we consider that the coverage graph is unweighted graph because we only study whether two nodes are connected, which is relative with the distance between those. If we study the energy consumption of sensors, the coverage graph can be assigned weight and the meaning of the edges’ weight is the energy. If the sensors are heterogeneous, the edges’ weights are different. In a word, we can give the weight different meanings according to the actual application background. Thus, the MNHG framework has wider application.

## 5. Simulation Results

In this section, simulations are conducted to study the performance of 1-MNHG algorithm and *k*-MNHG algorithm. The performance of two proposed algorithms, the shortest path algorithm [29] and the UT-adjacency matrix-based algorithm [30], are compared by constructing the strong barrier coverage.

In our simulations, *N* sensors are randomly deployed in the UWSN following a uniform distribution in a belt region of 10,000 × 500 $m^{2}$. Underwater environmental parameters are described in Table 1. Working frequency *f* is 6kHz. Under the conditions of 3 level sea state, SNL is about 46 dB. Receiving bandwidth $B_{f}$ is 500 Hz. 500 random topologies of UWSNs are generated to study the performance of the proposed algorithms. The simulation results are the average numbers of constructing barriers in 500 UWSNs. We assume that the sensors in UWSNs can communicate with each other. The energy attenuation disk coverage model in the UWSN is utilized to achieve the barrier coverage. Therefore, the deterministic sensing radius does not exist. $R_{s}$ is no longer the sensing radius and only expresses the radius used to construct barriers, which we call constructing radius. We assume that one target crosses the belt region along an arbitrary path.

### 5.1. Number of Comparisons of Sensors Constructing Strong Barrier Coverage

**Figure 9:**
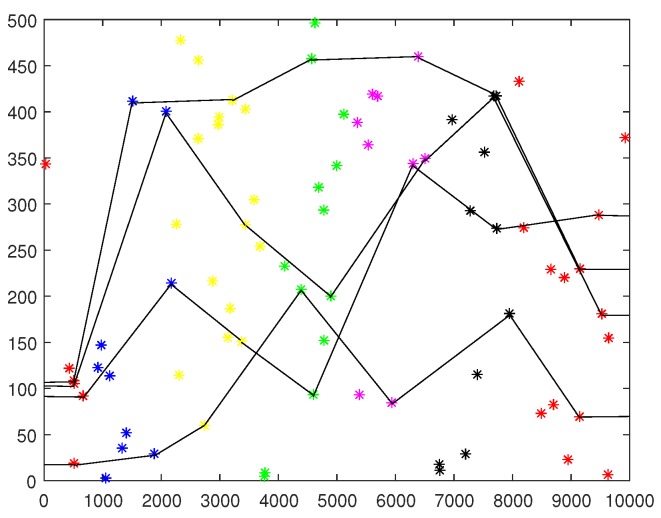
A UWSN constructed barriers with *k*-MNHG algorithm, when N=80 sensors are randomly deployed in the belt region.

In deterministic deployment, the minimum number of sensors constructing 1-barrier is $N_{opt}=\left\lceil \frac{L}{2R_{S}}\right\rceil$, which is the optimal number of sensors constructing 1-barrier in random deployment. Nevertheless, the optimal number is difficult to be achieved in random deployment. Figure 9 shows an UWSN that the barriers is constructed with the *k*-MNHG algorithm, when N=80 sensors are deployed randomly in the belt region and the constructing radius is 800 m. The sensors in different hierarchies are marked different colors. From Figure 9, we can see that this UWSN is graded to seven hierarchies.

**Figure 10:**
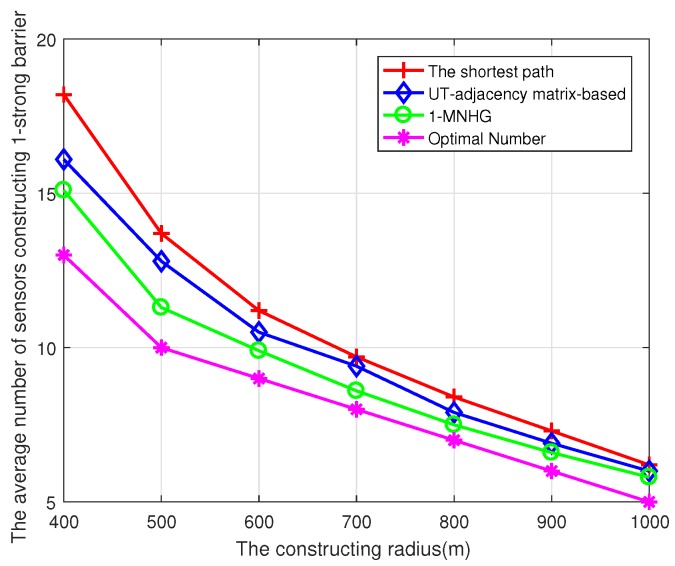
Compare the average number of sensors constructing a 1-strong barrier using three algorithms with different radii, when N=80 sensors are randomly deployed in the belt region.

In Figure 10, the comparison of the number of sensors constructing 1-strong barrier using several algorithms is made. With the increase of the constructing radius, the average number of sensors constructing 1-strong barrier using three algorithms are all decreased. The average number adopting 1-MNHG algorithm is minimal, and the average number using the shortest path algorithm is maximal. This is because the shortest path algorithm does not pursue the minimum number of required sensors, but the shortest path. Furthermore, the greater the number of sensors randomly deployed, the closer the gap between the average numbers of sensors for 1-strong barrier and the desired number. The main reason is that the probability of constructing barriers with a minimum number of sensors increases when the constructing radius and the number of deployed sensors in a UWSN increase. When the constructing radius is sufficiently large, the number of sensors constructing a 1-barrier using a 1-MNHG algorithm is close to the desired value. However, limited by random deployment, the desired value will be never reached.

**Figure 11:**
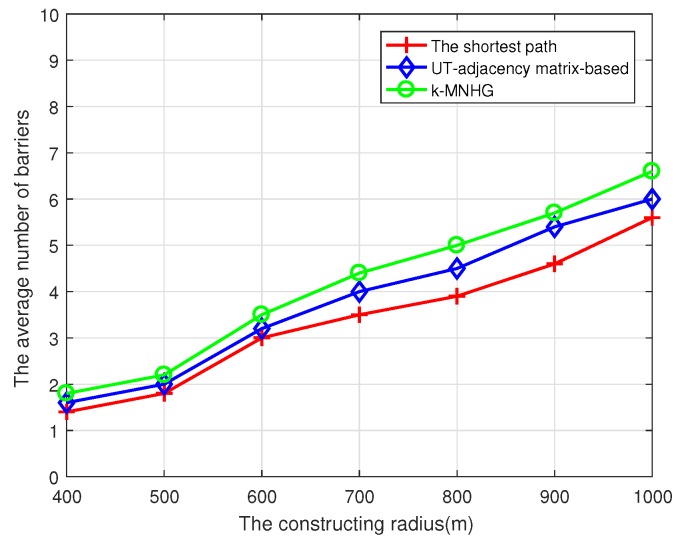
Compare the average number of strong barriers using three algorithms with different constructing radii when N=80 sensors are randomly deployed in the belt region.

Figure 11 shows that the comparison of the average number of constructed barriers using the *k*-MNHG algorithm and the other two algorithms with different constructing radii. It can be observed that, as the radius increases, the average number of constructed barriers using three algorithms increases. Furthermore, the number of barriers adopting *k*-MNHG algorithm is larger than that using the other two algorithms.

**Figure 12:**
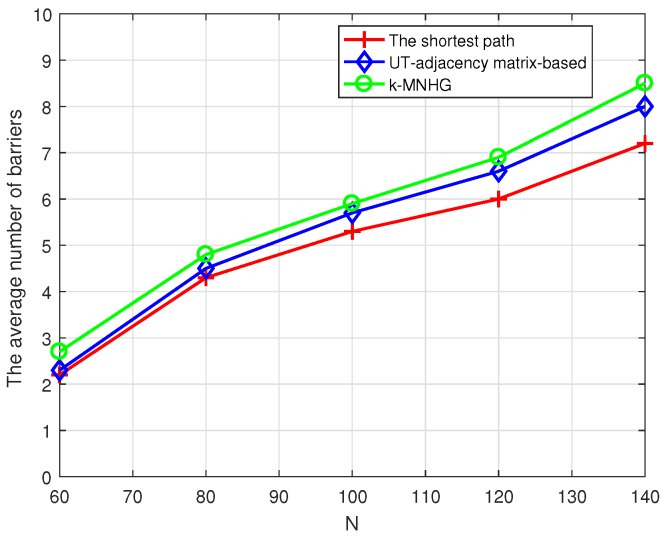
Compare the average number of strong barriers using three algorithms in different numbers of sensors randomly deployed, when the constructing radius is 800 m.

Figure 12 presents the comparison of the average number of barriers in different numbers of sensors randomly deployed, when the constructing radius is 800 m. The average number of constructed barriers using three algorithms increases with the increase of the number of sensors randomly deployed. In particular, the average number of constructed barriers using *k*-MNHG algorithm is always the largest.

**Figure 13:**
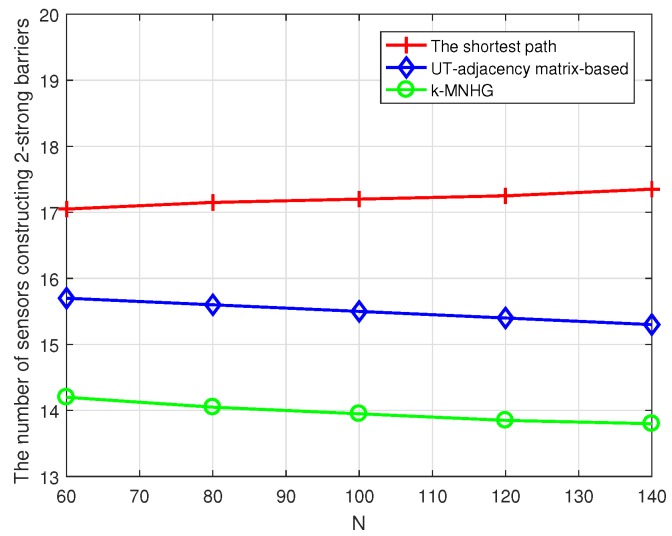
Compare the average number of strong barriers using three algorithms with different numbers of sensors randomly deployed, when the constructing radius is 1000 m.

Figure 13 shows that the comparison of the number of required sensors in different numbers of sensors randomly deployed, when only two strong barriers are constructed and the constructing radius is 800 m. The increase of the number of sensors randomly deployed has little impact on the number of required sensors. Figure 13 manifests that, when the same number of barriers are constructed, the number of required sensors constructing two strong barriers using the *k*-MNHG algorithm is the smallest and the number using the shortest path algorithm is the largest.

In general, in random deployment, the 1-MNHG algorithm and *k*-MNHG algorithm can be adopted to achieve barrier coverage with less sensors than the other two algorithms, which is closest to the desired number.

### 5.2. Comparison of UWSNs’ Detection Probability

**Figure 14:**
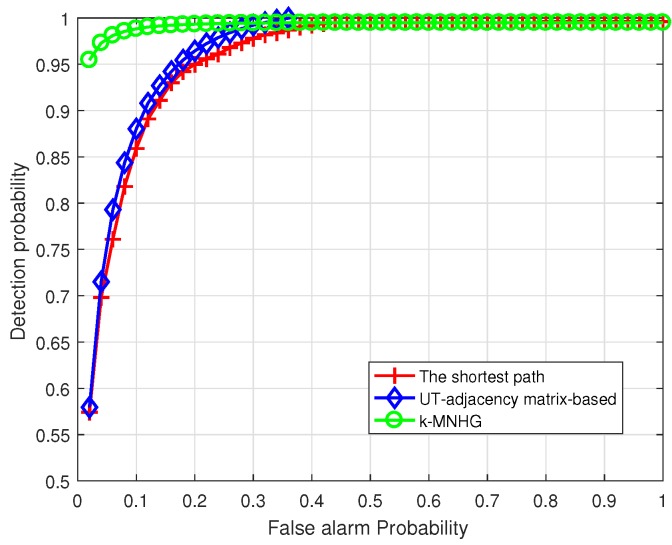
Compare the ROC curve of the systems using three algorithms, when SL = 145 dB and the constructing radius is 800 m.

**Figure 15:**
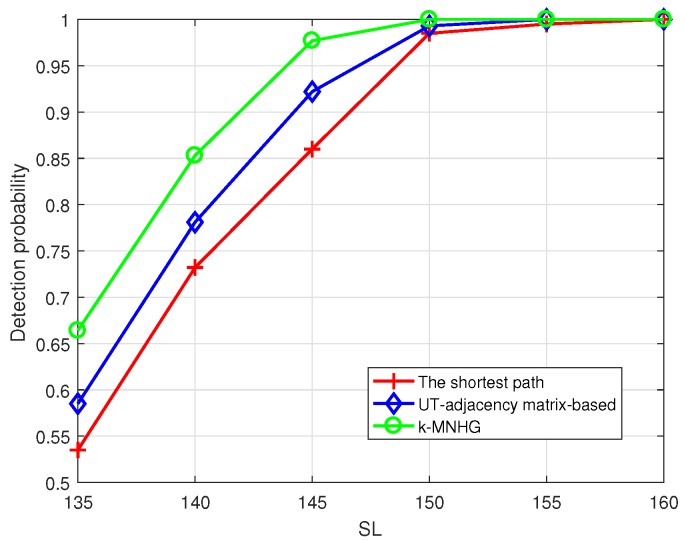
Compare the detection probability of the systems using three algorithms in different SL, when the constructing radius is 800 m and the false alarm probability $P_{f}$ = 0.1.

**Figure 16:**
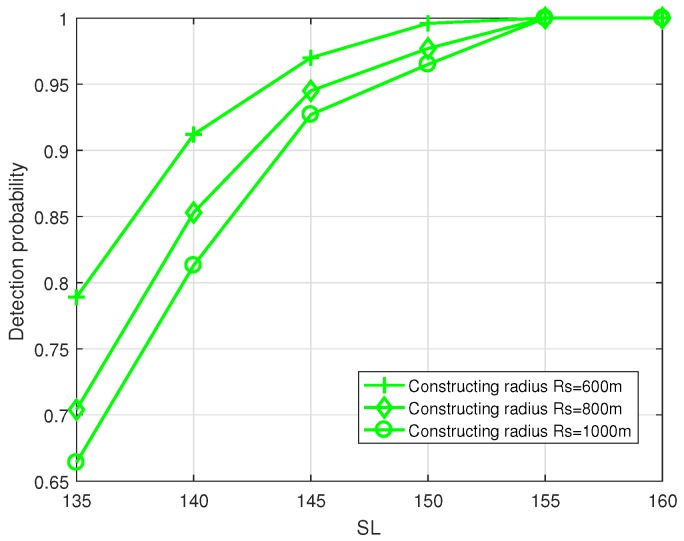
Compare the detection probability of the system using *k*-MNHG algorithm with different constructing radius, when the false alarm probability $P_{f}$ = 0.1.

Figure 14, Figure 15 and Figure 16 compare the UWSNs’ detection probability adopting the *k*-MNHG algorithm, the shortest path algorithm and the UT-adjacency matrix-based algorithm, respectively, when N=80 sensors are randomly deployed in the belt region. In the simulations below, we assume that all the barriers work at the same time. We detect the target adopting the Neyman–Pearson criterion: maximizing the detection probability under certain false alarm probability. The number of Monte Carlo simulations is $K=10^{4}$.

From Figure 14, we can see that, when SL = 145 dB and the constructing radius is 800 m, the receiver operating characteristic(ROC) curve of the systems adopting the *k*-MNHG algorithm is always higher than 95%. Additionally, the detection probability of the system using the shortest path algorithm to construct the *k*-strong barrier is the lowest and that using the *k*-MNHG algorithm to construct *k*-strong barrier is the highest. This is because the number of barriers using the shortest path algorithm is the smallest and the number of barriers using the *k*-MNHG algorithm is the largest, which has been shown in Figure 11.

Figure 15 shows that the detection probability of the systems using three algorithms can reach 95%, when SL = 150 dB, $P_{f}$ = 0.1 and the constructing radius is 800 m. The detection probability of the system using *k*-MNHG algorithm can reach to 95% when SL = 145 dB, which is higher than the other two algorithms.

Figure 16 shows that, the larger the radius, the lower the detection probability of the system. Ensuring the detection probability of the system 95% when SL = 145 dB, the effective sensing radius using *k*-MNHG algorithm is 600 m.

### 5.3. Comparison of UWSNs’ Lifetime

**Figure 17:**
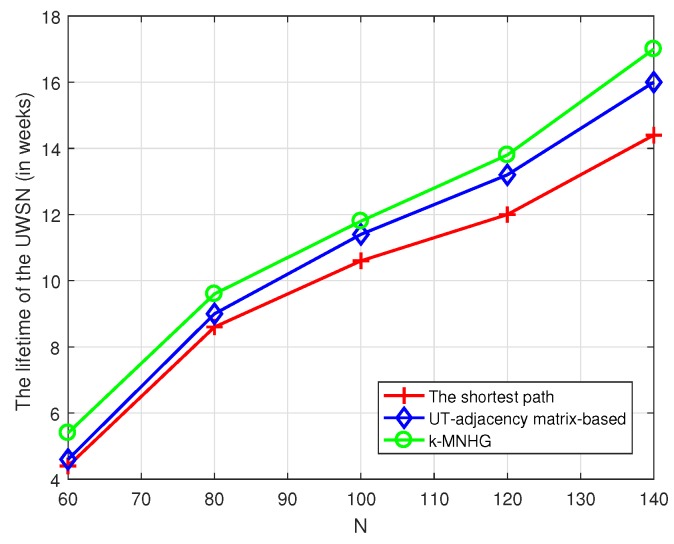
Compare the UWSNs’ lifetime using three algorithms with different numbers of sensors randomly deployed, when all the barriers take turns working and the constructing radius is 800 m.

For the homogeneous case, we assign a lifetime of two weeks for each sensor. Moreover, we assume that all the barriers take turns working. In other words, there is only one barrier working every time. In Figure 17, we compare the lifetime of UWSNs using three algorithms. The comparison result is similar to Figure 11, owing to the number of barriers being in direct relation to the sensor network lifetime. From this figure, we can see that the lifetime using three algorithms increases with the increase of the number of sensors randomly deployed in UWSNs. Furthermore, the lifetime using *k*-MNHG is the longest.

In conclusion, the minimum number of sensors for 1-barrier using an 1-MNHG algorithm is smaller than the other two algorithms. In addition, the computational complexity of 1-MNHG algorithm is lower than the other two algorithms. Moreover, the *k*-MNHG algorithm is superior to the shortest path algorithm and the UT-adjacency matrix-based algorithm in relation to high detection probability and long lifetime. Furthermore, the detection probability of the system using the proposed *k*-MNHG algorithm can reach 95%, when SL = 145 dB and *N* = 80 sensors are randomly deployed in the belt region, which proves that the barrier coverage strategy based on a hierarchy graph is more suitable for UWSNs.

## 6. Conclusions

In this paper, we study the barrier coverage strategy in random deployment and make two main contributions. Firstly, a new concept: hierarchy graph is introduced to construct barriers. Based on a hierarchy graph, the barrier coverage with a minimum number of stationary sensors is obtained. Secondly, two algorithms: (i) 1-MNHG algorithm and (ii) *k*-MNHG algorithm are proposed. Our simulation results show that the barrier coverage strategy based on hierarchy graph can construct barriers with less sensors, which improves the detection probability and prolongs UWSNs’ lifetime. Moreover, the computational complexity of the new strategy is not increased. In addition, by assigning the weight of the hierarchy graph different meanings and different value, the proposed algorithms in this paper can be applied to a broad range of applications.

A new coverage strategy in this paper is proposed based on the graph theory. The constructing barrier algorithms based on graph theory depends on the sensors’ location information strongly. In the future, we will study the hole monitoring and filling strategy based on the hierarchy graph. This strategy can monitor the sensors’ locations and find the hole to fill it. Moreover, we will consider utilizing the cooperative detection technique, which can indirectly increase sensors’ sensing radii. Based on the cooperative detection technique, we can construct more barriers using less sensors.

## Figures and Tables

**Table 1:** Parameter description.

Parameter	Value
1. Belt region’s area	10,000 $\times 500\;m^{2}$
2. Noise spectrum level over 1Hz bandwidth, SNL	46 dB
3. Receiving directivity index (DI)	0
4. Receiving bandwidth ($B_{f}$)	500 Hz
5. Working frequency (*f*)	6 kHz

## Abbreviations

The following abbreviations are used in this manuscript:

UWSNs	Underwater sensor networks
WSNs	Wireless sensor networks
MNHG	Obtain barriers with a minimum number of required sensors based on hierarchy graph
SL	Source level
TL	Transmission loss
NL	Noise level
SNL	Noise spectrum level over 1 Hz bandwidth
$B_{f}$	Receiving bandwidth
SNR	Signal-to-noise ratio
